# Subpolar North Atlantic western boundary density anomalies and the Meridional Overturning Circulation

**DOI:** 10.1038/s41467-021-23350-2

**Published:** 2021-05-24

**Authors:** F. Li, M. S. Lozier, S. Bacon, A. S. Bower, S. A. Cunningham, M. F. de Jong, B. deYoung, N. Fraser, N. Fried, G. Han, N. P. Holliday, J. Holte, L. Houpert, M. E. Inall, W. E. Johns, S. Jones, C. Johnson, J. Karstensen, I. A. Le Bras, P. Lherminier, X. Lin, H. Mercier, M. Oltmanns, A. Pacini, T. Petit, R. S. Pickart, D. Rayner, F. Straneo, V. Thierry, M. Visbeck, I. Yashayaev, C. Zhou

**Affiliations:** 1 State Key Laboratory of Marine Environmental Science & College of Ocean and Earth Sciences, Xiamen University, Xiamen, China; 2 School of Earth and Atmospheric Sciences, Georgia Institute of Technology, Atlanta, GA USA; 3 National Oceanography Centre, Southampton, UK; 4 Woods Hole Oceanographic Institution, Woods Hole, MA USA; 5 Scottish Association for Marine Science, Oban, UK; 6 NIOZ Royal Netherlands Institute for Sea Research, Texel, Netherlands; 7 Department of Physics and Physical Oceanography, Memorial University, St. John’s, NL Canada; 8 Fisheries and Oceans Canada, Institute of Ocean Sciences, Sidney, BC Canada; 9 Fisheries and Oceans Canada, Northwest Atlantic Fisheries Centre, St. John’s, NL Canada; 10 Scripps Institution of Oceanography, UCSD, La Jolla, CA USA; 11 School of Geosciences, Edinburgh University, Edinburgh, UK; 12 Department of Ocean Sciences, University of Miami, Miami, FL USA; 13 GEOMAR Helmholtz Centre for Ocean Research Kiel, Kiel, Germany; 14 Univ. Brest, Ifremer, CNRS, IRD, Laboratoire d’Océanographie Physique et Spatiale, Plouzané, France; 15 Frontier Science Center for Deep Ocean Multispheres and Earth System and Physical Oceanography Laboratory, Ocean University of China, Qingdao National Laboratory for Marine Science and Technology, Qingdao, China; 16 CNRS, Laboratoire d’Océanographie Physique et Spatiale, Plouzané, France; 17 Bedford Institute of Oceanography, Dartmouth, NS Canada

**Keywords:** Ocean sciences, Physical oceanography

## Abstract

Changes in the Atlantic Meridional Overturning Circulation, which have the potential to drive societally-important climate impacts, have traditionally been linked to the strength of deep water formation in the subpolar North Atlantic. Yet there is neither clear observational evidence nor agreement among models about how changes in deep water formation influence overturning. Here, we use data from a trans-basin mooring array (OSNAP—Overturning in the Subpolar North Atlantic Program) to show that winter convection during 2014–2018 in the interior basin had minimal impact on density changes in the deep western boundary currents in the subpolar basins. Contrary to previous modeling studies, we find no discernable relationship between western boundary changes and subpolar overturning variability over the observational time scales. Our results require a reconsideration of the notion of deep western boundary changes representing overturning characteristics, with implications for constraining the source of overturning variability within and downstream of the subpolar region.

## Abstract (web summary)

Western boundary current variability in the subpolar North Atlantic is thought to reflect interior convection changes and determine Atlantic Meridional Overturning Circulation variability. Here, the authors show with an extended OSNAP time series that neither linkage is robust due to the complex dynamics in the region.

## Introduction

The high-latitude North Atlantic is a key region in the global ocean circulation system. Strong buoyancy loss creates North Atlantic Deep Water (NADW) that subsequently spreads to other ocean basins via the Deep Western Boundary Current (DWBC)^1,2^ and interior (as opposed to DWBC) pathways^3^. The formation and spreading of NADW are essential elements of the Atlantic Meridional Overturning Circulation (MOC)^4,5^. Paleoclimate studies have suggested a strong association between abrupt climate changes during the last glacial cycle and changes in MOC strength, the latter attributed to the strength and location of deep water formation in the subpolar region^6^. On modern time scales, the cessation of deep water formation by winter convection in the Labrador Sea has been proposed as a potential tipping point for future climate change^7^. Moreover, a recent study has suggested an emerging impact of increased freshwater export on weakened deep water formation in the subpolar region^8^. Thus, deciphering relationships between the formation and export of deep water and the MOC’s structure as well as variability is of central importance to understanding and predicting the effects of a warming climate.

**Fig. 1:**
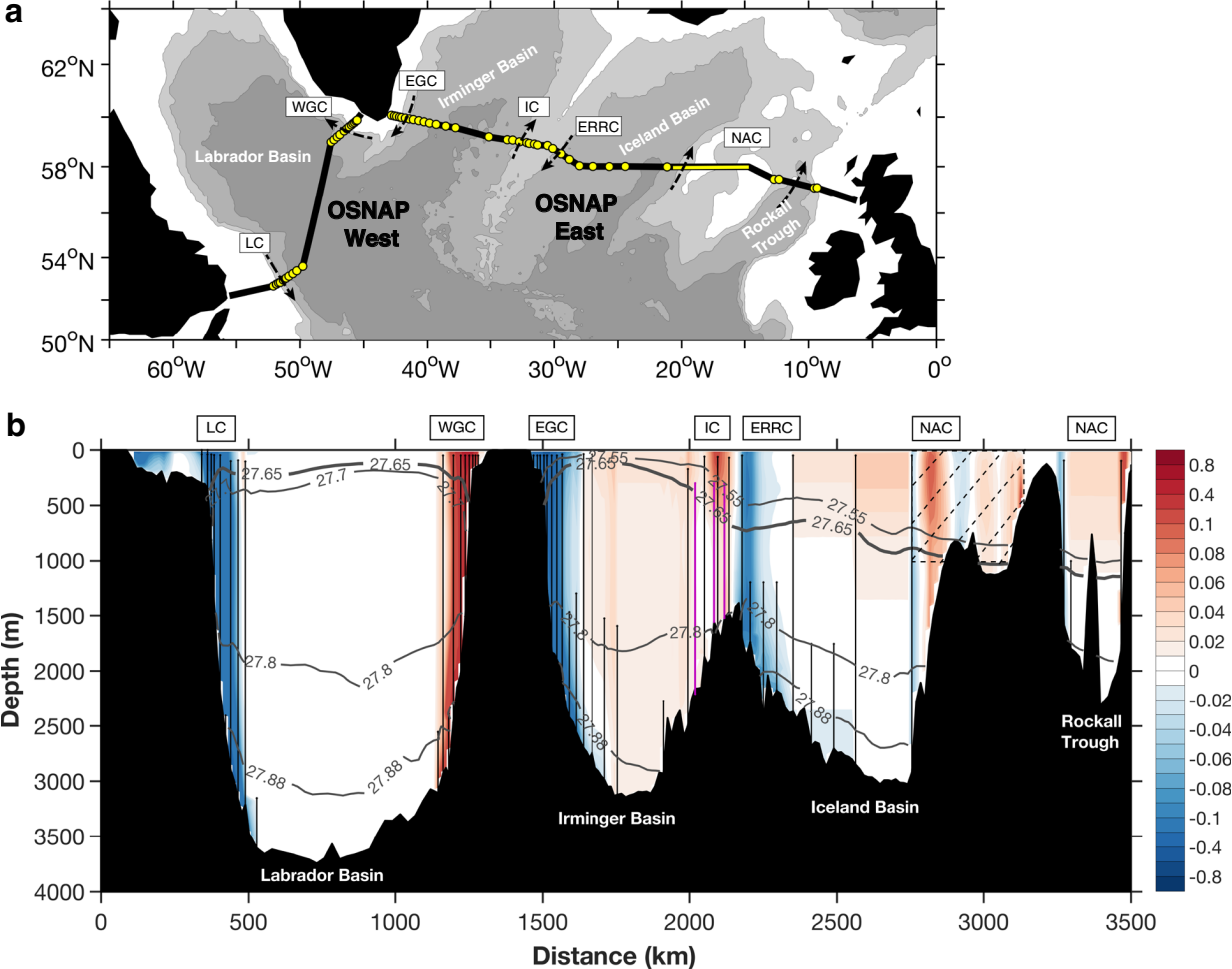
OSNAP array. **a** Locations of OSNAP moorings (yellow dots) and glider survey domain (yellow line) on bathymetry (1000 m intervals). Arrows indicate the major currents intercepted by the OSNAP array from west to east: LC Labrador Current, WGC West Greenland Current, EGC East Greenland Current, IC Irminger Current, ERRC East Reykjanes Ridge Current, NAC North Atlantic Current. **b** 2014–2018 Mean velocity perpendicular to the OSNAP section (units: m s^−1^; positive poleward), overlaid by isopycnals (contoured). The isopycnal of 27.65 kg m^−3^ delimits the upper and lower limbs of the subpolar Meridional Overturning Circulation (MOC), which is slightly different for subsections (27.70 and 27.55 kg m^−3^ for OSNAP West and East respectively). The OSNAP moorings are marked by vertical black lines. Three moorings from the French Reykjanes Ridge Experiment (RREX) program are marked by vertical purple lines. Hatching in the eastern Iceland basin indicates the glider survey domain.

Recent results from a new trans-basin ocean observing system in the subpolar North Atlantic (OSNAP; Fig. 1)^9,10^ showed that water mass transformation in the eastern subpolar gyre (east of Greenland) dominated subpolar overturning over the period from 2014 to 2016. Surprisingly, winter convection in the Labrador Sea contributed minimally to the mean and variability of the subpolar MOC, even though unusually strong convection occurred in that basin during winter 2014/2015^11,12^. These results contradict the view of convection in the Labrador Sea as the major contributor to MOC variability throughout the North Atlantic^13,14^ via the propagation of density anomalies created by the varying strength of deep convection in this basin^15–19^. Though models disagree as to the strength of the linkage between Labrador Sea convection and the MOC, this linkage is a consistent model feature^20^. These new observations raise the question about the source of Labrador Sea density anomalies and their impact on MOC variability in the subpolar basin.

Because there is the possibility of a delayed impact of strong convection on the overturning due to a residence time of ~2–3 years for newly-formed upper NADW (UNADW) in the Labrador Sea interior^21,22^, there is a valid argument that the initial OSNAP record, 21 months in duration, was insufficient to capture this impact. Thus, in this study we use the extended OSNAP data between August 2014 and May 2018 (46 months) now available to further examine the linkage between wintertime convection and MOC variability. Namely, we first assess the impact of subpolar convection on western boundary density anomalies and then, in turn, quantify the impact of those western boundary anomalies on the subpolar MOC variability. We analyze OSNAP array observations to assess this variability on monthly to interannual time scales and define the MOC as the maximum of the overturning stream function in density (*σ*_*θ*_) space (Supplementary Fig. 1). Details of the OSNAP array design and calculations can be found in Methods.

## Results

### Subpolar overturning circulation

**Fig. 2:**
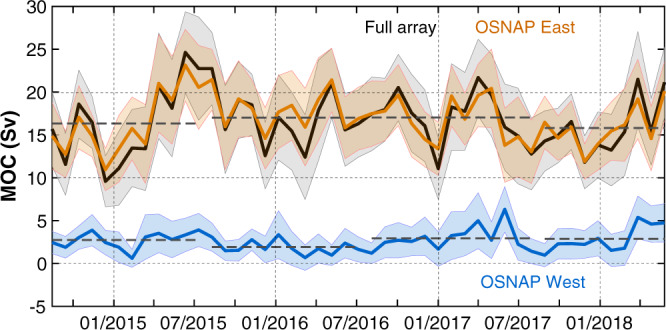
Subpolar Meridional Overturning Circulation (MOC) time series. 30-day MOC estimates across the full array, OSNAP West and East, respectively. Shading indicates uncertainty in the 30-day estimate, obtained from a Monte Carlo method^10^. Horizontal dashed lines indicate the 12-month averages (10-month for 2017–2018). The total Ekman transport (not shown) is $-1.5\,\pm\, 0.02\,{{{\rm{Sv}}}}$ during the whole time period.

Over our study period, the MOC across the OSNAP array has a time-mean of $16.6\,\pm\, 0.7\,{{{\rm{Sv}}}}$ ($1\,{{{\rm{Sv}}}}\,=\,\,{10}^{6}\,{{{{\rm{m}}}}}^{3}\,{s}^{-1}$) and exhibits strong monthly variability overlaid by year-to-year differences (Fig. 2). The quoted uncertainty is the standard error in the mean, estimated via Monte Carlo simulations based on the mean and uncertainty in individual months^10^. Although the 2014–2018 mean MOC is larger than the 2014–2016 mean^10^, the difference is not statistically significant at the 95% level (Methods). Composite monthly means, constructed by averaging the values of the same month for all years, appear to show seasonal MOC cycles (Supplementary Fig. 2), but the seasonal changes are not statistically significant (Methods).

On monthly to interannual time scales, MOC variability is dominated by overturning in the eastern subpolar gyre (OSNAP East) rather than in the Labrador Sea (OSNAP West). OSNAP East explains 82% of the total MOC variance and its mean ($16.8\,\pm\, 0.6\,{{{\rm{Sv}}}}$) is approximately seven times larger than the mean for OSNAP West ($2.6\,\pm\, 0.3\,{{{\rm{Sv}}}}$). Finally, we note that for this 4-year period, there is no evidence of a delayed MOC response to the intense Labrador Sea convection in winter 2014/2015. Therefore, the results confirm the dominance of overturning in the eastern subpolar gyre over that in the Labrador Sea, and confirm the weak response of overturning to strong Labrador Sea convection, both reported in the earlier analysis^10^.

### Linkage between deep convection and western boundary changes

We further investigate a linkage, or lack thereof, between winter convection and the subpolar MOC by first focusing on the impact of deep convection in the interior on UNADW thickness anomalies along the western boundaries of the Labrador Sea and Irminger Sea. Here we use UNADW layer thickness, defined by the vertical distance between two density surfaces, as a proxy for density anomalies in the boundary and basin interior^23^. The thickness anomalies are derived relative to their 46-month mean. The upper isopycnal of UNADW is the density surface associated with the MOC, which is 27.70 and 27.55 kg m^−3^ for OSNAP West and East, respectively (Fig. 1b, Supplementary Fig. 1). The lower isopycnal for UNADW at both sections is 27.80 kg m^−3^, chosen to exclude the lower overflow component of NADW (LNADW). We refer to UNADW and LNADW as the water masses contained in the lower limb of the MOC (e.g.,^3,24^). For the thickness calculation in the basin interior, we add a planetary potential vorticity constraint (<4 × 10^−12^ m^−1^ s^−1^) to identify newly-formed deep waters^25^. The expected signature of water mass transformation in the Labrador and Irminger Seas during winter convection is an increase in UNADW layer thickness, as lighter surface water cools, slightly freshens and loses buoyancy^11,26^.

**Fig. 3:**
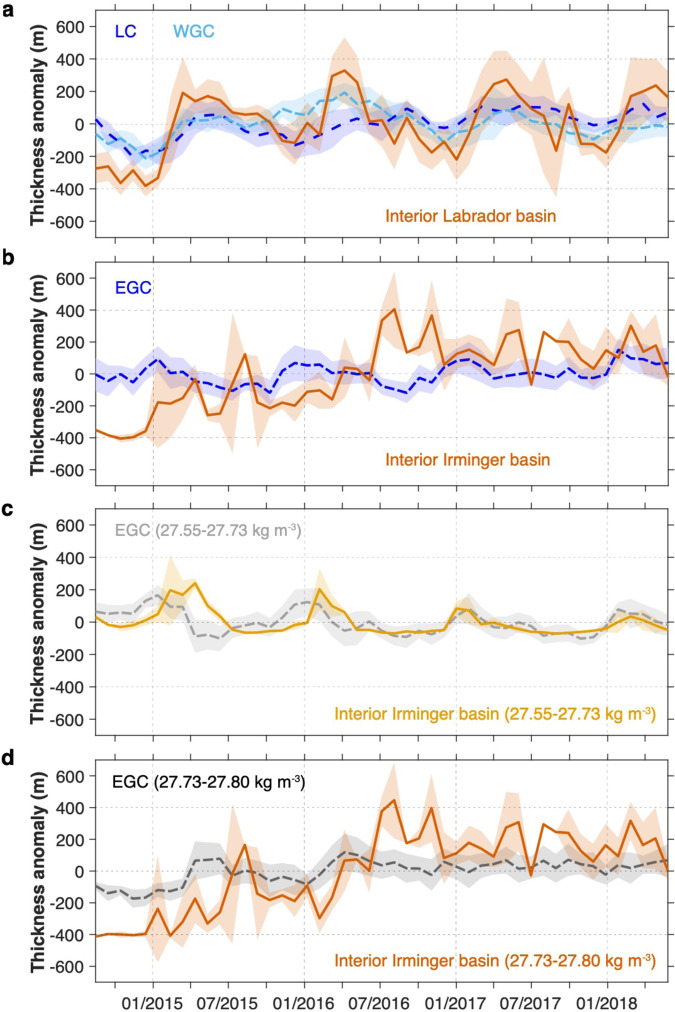
Upper North Atlantic Deep Water (UNADW) layer thickness anomalies. **a** Labrador Sea: UNADW layer (*σ*_*θ*_ = 27.70–27.80 kg m^−3^) thickness anomalies across the full Labrador Current (LC; dark blue) and West Greenland Current (WGC; light blue) arrays (see Fig. 1b for location), respectively, with shading represents uncertainty; layer thickness anomalies in the Labrador Sea interior (red, shading represents ±1 standard deviation) computed from Argo data north of the OSNAP line where seafloor >3000 m deep (Methods). **b** Irminger Sea: UNADW (*σ*_*θ*_ = 27.55–27.80 kg m^−3^) layer thickness anomalies across the East Greenland Current (EGC) array of tall moorings within the boundary current (blue; see Fig. 1b for location), with shading represents uncertainty; layer thickness anomalies in the Irminger Sea interior (red, shading represents ±1 standard deviation) computed from Argo data north of the OSNAP line where seafloor >2000 m deep (Methods). **c**, **d** Irminger Sea: similar as in (**b**), but for the lightest (*σ*_*θ*_ = 27.55–27.73 kg m^−3^) and most dense UNADW layers (*σ*_*θ*_ = 27.73–27.80 kg m^−3^), respectively.

UNADW layer thickness exhibits clear seasonality in the Labrador Sea interior, increasing by at least 500 m from January to April of each year (Fig. 3a). These changes are consistent with the characteristics of UNADW formation and ventilation on seasonal to interannual time scales^11,27^. UNADW layer thickness in the Labrador Sea boundary currents (Labrador Current, LC and West Greenland Current, WGC, Fig. 1) shows similar variability, but is less than half the magnitude observed in the interior. This boundary-interior difference in thickness change is related to a number of factors: the comparatively weak water mass transformation in the boundary current^28^ (Supplementary Fig. 3); the compensating exchange of temperature and salinity anomalies between the interior and the boundary current^29^; and the fact that the interior-boundary exchange occurs over time scales from days to years^30,31^.

The import of upstream thickness anomalies may also obscure the relationship between thickness anomalies in the interior and the boundary currents. For example, the increase in WGC thickness in late 2015 and early 2016 precedes thickness increases in the LC and Labrador Sea interior, suggesting a possible upstream source. To support this supposition, we point to the similarity of the LC, WGC, and East Greenland Current (EGC) anomaly time series (blue lines in Fig. 3a, b). Using daily values at these three boundary arrays, lagged correlations show that the maximum correlation (significant at the 95% level) occurs when the EGC thickness leads the WGC thickness by 22 days and the WGC thickness leads LC thickness by 310 days (Methods). These lags are consistent with a signal propagating via the boundary current system at the advective speed ~10 cm s^−1^ around Greenland^32^ and the Labrador basin^30,31^. However, we also note the shared seasonality in these time series, with significant positive correlations at near zero lag and significant negative correlations at ~±180 days. Thus, in addition to the advective mechanism, it is also plausible that the observed ~1-year lag between the EGC and LC is a signature of a common seasonal cycle. A longer time series will be needed to carefully differentiate these possibilities.

In the Irminger Sea a different picture emerges. While the UNADW thickness in the Irminger Sea interior exhibits strong monthly and interannual variability, including a sustained thickening during 2015–2016, a seasonal signal is not evident (Fig. 3b). In contrast, UNADW in the EGC exhibits clear seasonality, but weak interannual variability. Pulses of thick UNADW appear every January in this boundary current, the strongest of which are in the early record. To further examine this dissimilarity between the thickness variability in the interior and boundary current, we partition the UNADW into its light and dense components. With this partitioning, thickness changes in the interior and in the EGC are now comparable for the lightest UNADW (27.55–27.73 kg m^−3^; Fig. 3c), with strong seasonality in both time series. The boundary current thickness has an earlier peak most winters, with wintertime thickening in both time series diminishing over the observational record (from ~200 m in 2014/2015 to ~100 m in 2017/2018). It appears that this light UNADW component can be formed within the EGC itself, and also rapidly exported into the EGC from the interior, as previously reported from an analysis of OSNAP data^33^.

Changes for the dense UNADW layer (27.73–27.80 kg m^−3^) in the interior mirror those for the full UNADW layer (Fig. 3d). The gradual thickening during 2015–2016 may be associated with deep convection in the southwest Irminger basin^12,34,35^ or the arrival of newly-formed UNADW from the Labrador basin, estimated to reach the Irminger basin via an interior pathway in ~1/2–2 years^22,36^. Thickness anomalies in the EGC for the dense UNADW are evident for the winters of 2014/2015 and 2015/2016 (Fig. 3d).

Our analysis has shown that only for the light UNADW in the Irminger Sea is there a simple relationship between thickness changes in the basin interior and the boundary currents. Thus, as with the Labrador Sea, we expect that thickness changes in the boundary current can be impacted by convection within the boundary current, by along-stream advection of thickness anomalies, as well as by convection in the interior. Similarly, as suggested above, it is likely that some thickness anomalies in the interior are also imported. Collectively, these impacts create records of interior and boundary variability that preclude clear attribution and linkage at least on the time scales studied here.

### Linkage between western boundary changes and the MOC

We next evaluate the extent to which UNADW thickness anomalies in the OSNAP boundary arrays impact subpolar MOC strength. As has been shown previously, thickness variability in these boundary currents determines volume transport variability over seasonal and longer time scales^32,33,37^. In the Labrador Sea, thickness in the LC and WGC largely co-vary with time with small differences between them (~300 m). The thickness difference is related to the strength of the overturning because it reflects the change in density gradients across the basin. This change impacts the vertical velocity shear and thus the geostrophic flow carrying UNADW out of the Labrador Sea. In other words, the relatively low amplitude of the OSNAP West MOC variability arises from the cancellation of thickness changes in the boundary currents on either side of the Labrador Sea. Assuming a linear relationship between the layer thickness and the MOC, the layer thickness difference between the two boundaries would need to increase more than threefold (by ~900 m) for the OSNAP West MOC magnitude to reach that of OSNAP East (16.8 Sv). Such a change in the layer thickness far surpasses that observed in the basin during the past couple of decades^26^. When considering the nonlinearities of the system (e.g., the impact of layer thickness change on the velocity through the modification of the baroclinic shear in the boundary current), an even larger difference in the layer thickness would be needed to produce the same MOC increase.

**Fig. 4:**
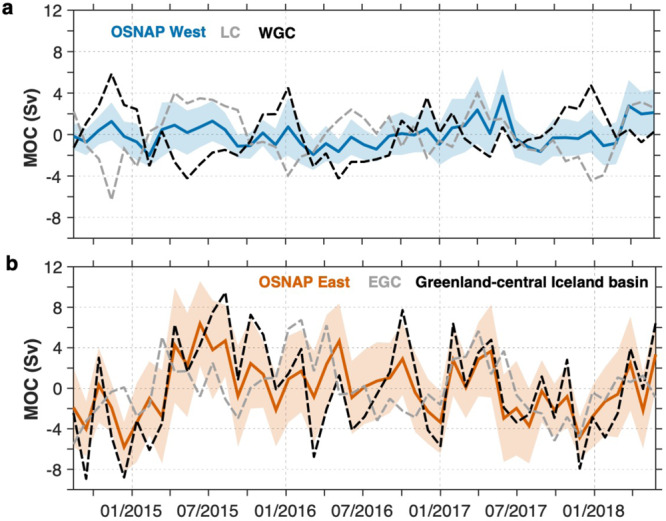
Components of overturning variability. **a** OSNAP West Meridional Overturning Circulation (MOC) anomalies: overturning derived from the Labrador Sea array (blue; shading indicates uncertainty as shown in Fig. 2); MOC variability arising from time-varying density and velocity anomalies in the Labrador Current (LC; light gray) computed with time-mean velocities/densities at the West Greenland Current (WGC) boundary, and MOC variability arising from density and velocity anomalies in the WGC (black) computed with time-mean velocities/densities at the LC boundary. **b** OSNAP East MOC anomalies: overturning derived from the OSNAP East array (red; shading indicates uncertainty as shown in Fig. 2); MOC variability arising from time-varying density and velocity in the region between Greenland and mid-Iceland basin (black) computed with time-mean velocities/densities at the eastern boundary, and MOC variability arising from density and velocity anomalies in the East Greenland Current (EGC; light gray) computed with time-mean velocities/densities everywhere else. For the reconstruction based on the time-varying data at the western boundary (light gray line), the MOC is defined as the minimum of the stream function integrated from the bottom to the sea surface in density space (sign has been changed for comparisons).

A calculation of OSNAP West MOC based only on LC and WGC velocity and density fields captures ~70% of the MOC variance calculated using data across the full OSNAP West section (${r}^{2}\,\approx\, 0.7$; Methods). It is consistent with the importance of the boundary region for the Labrador Sea overturning^21,38^. To further separate the contributions from the LC and WGC boundaries, we compute the MOC using time-varying fields at one boundary array and time-mean fields at the other, and then vice versa. Although variance in the OSNAP West MOC produced by changes in the LC and WGC individually (~6 Sv^2^) exceeds the actual OSNAP West MOC variance (~2 Sv^2^), changes occurring on either side can explain no more than ~10% of the actual OSNAP West MOC variance (Fig. 4a). Furthermore, neither the anomalies in the individual boundary currents nor their combined effects have a statistically significant impact on the strength of the full subpolar MOC, as expected since the OSNAP West MOC contributes weakly to the full MOC.

Turning to the eastern subpolar gyre, we first note that the geometry of the overturning here is remarkably different from that in the Labrador Sea. The MOC upper limb is mainly constrained to the eastern part of the OSNAP East array, where the North Atlantic Current flows broadly northward, and the lower limb is largely constrained to the central and western portions of the array (Fig. 1b). However, density and velocity changes at the western (EGC) and eastern (Rockall Trough) boundaries together explain only ~50% of the total MOC variance derived from the full OSNAP East array. Changes in the EGC alone capture an even smaller fraction (10%) of the variability in the OSNAP East MOC (Fig. 4b). Thus, variability in the western boundary current of the Irminger basin is not an indicator of the overturning circulation in the eastern subpolar North Atlantic on the time scale of these observations. However, when we consider changes in a wider region between Greenland and the central Iceland basin (e.g., the location at the OSNAP line that approximately separates the upper and lower limbs of MOC; see Fig. 1b for the location ~2600 m), we are able to reconstruct ~75% of the total MOC variance across OSNAP East (Fig. 4b). Across OSNAP East, the MOC lower limb is captured only by including currents in a broad region that extends well beyond the western boundary current. The variability contained within the lower limb at OSNAP East is, of course, matched by variability in the upper limb; changes in the upper limb for the region between the central Iceland basin and Scotland explain ~65% of the full subpolar MOC (Supplementary Fig. 4).

## Discussion

The extended OSNAP time series has supported the weak linkage between Labrador Sea convection and the subpolar MOC, even during a period with pronounced changes in deep convection. Furthermore, our results demonstrate that density anomalies along the western boundaries of the Labrador and Irminger Seas are not exclusively determined by changes in their basins’ interior. We suggest that convection in the boundary current itself, along-stream advection of upstream anomalies, and the limited exchange between the interior and the boundary are collectively responsible for the differences in the records of variability. The light component of UNADW in the Irminger Sea is an exception to this description. We note also that the interiors of the basins are themselves subject to the import of anomalies created elsewhere in previous winters, as noted earlier for the Irminger Sea.

These OSNAP observations reveal that changes in the western boundary current in the subpolar North Atlantic are not, by themselves, indicators of subpolar MOC variability on monthly to interannual time scales. The MOC lower limb in the subpolar North Atlantic has a complex circulation^3,39^ and changes in the full set of pathways combine to describe the MOC and its variability. Thus, a partial measurement (or proxy) of the DWBC transport or density variations in a limited geographical area (e.g., in the central Labrador Sea or within the Labrador Sea western boundary) is insufficient to reconstruct MOC changes in the subpolar region on these time scales.

A recent, related study finds that the water mass transformation induced by air-sea heat and freshwater fluxes in the broad region between the Greenland-Scotland Ridge and OSNAP East can explain the overturning difference between those two sections (~7 Sv)^40^. Thus, the rapid increase in the MOC in the spring of 2015 can be attributed to strong buoyancy forcing throughout the eastern subpolar basin during the 2014/2015 winter. This distributed transformation is consistent with our analysis above in that it suggests the full OSNAP East array is needed to capture the full measure of the overturning.

In summary, our results cast further doubt on the supposition that convection variability in the central Labrador Sea drives MOC variability via the export and propagation of density anomalies along the western boundary. In support of our work here, a recent modeling study^41^ finds that density anomalies advected from the eastern subpolar North Atlantic dominate the density variability in the western boundary of the Labrador Sea. Thus, density anomalies found in the Labrador Sea likely carry upstream signals. Furthermore, other recent studies have suggested that the linkage between Labrador Sea convection and downstream MOC variability can be explained by shared variability in response to the North Atlantic Oscillation and other atmospheric forcing^42,43^, rather than by the equatorward propagation of MOC anomalies from the Labrador Sea.

The 4-year OSNAP record provides new insights into the characteristics of the subpolar overturning variability, in particular, its relationship to deep convection and variability at the western boundary. Because our analysis is limited to the time scales resolved by the available record, we acknowledge that the observed relationships may not hold over longer time scales. However, since recent modeling studies do not yet agree on the role of the Labrador Sea in driving overturning variability on decadal and longer time scales (e.g.,^41,44^), longer direct measurements are clearly needed. OSNAP is an ongoing program and aims to provide at least 10 years of continuous observations in the region.

## Methods

### MOC calculations

Here we provide a brief summary of the MOC definition at OSNAP. For more details on calculations of the property and velocity fields as well as the MOC and its uncertainty, the reader is referred to Li et al.^45^ and Lozier et al.^10^.

MOC is defined as the maximum of the overturning stream function in *σ*_*θ*_ space, Ψ, as:

1 $${{{\rm{MOC}}}}(t)\,=\,\,{{\max }}[\Psi ({{\sigma }},{{{\rm{t}}}})]\,=\,\,{{\max }}\left[\int _{{\sigma }_{{{\min }}}}^{\sigma }\int _{{x}_{w}}^{{x}_{e}}v(x^{\prime} ,\sigma^{\prime} ,\,t)\,dx^{\prime} d\sigma^{\prime} \right]\,({{{\rm{Sv}}}}),$$

where *v* is the volume transport per unit length per unit density and is perpendicular to the OSNAP section (poleward positive). The double integrals are taken from west (*x*_*w*_) to east (*x*_*e*_) and from the top (*σ*_min_) across all density surfaces. The MOC upper (lower) limb is defined as the transport between the surface (bottom) and the density at which the overturning function reaches a maximum (namely *σ*_MOC_). The 2014–2018 mean *σ*_MOC_ is $27.65\,{{{\rm{kg}}}}\,{m}^{-3}$ across OSNAP (Fig. 1b).

### Updates to the MOC calculations

There have been a couple of updates in the calculations since Lozier et al.^10^ which are related to changes in the array configuration (e.g., addition or loss of instruments) or in the auxiliary data products. Those changes affect the calculations of the cross-sectional velocity field, and, to a lesser extent, the MOC estimate when integrating over the whole section. Here we describe individual changes generally from west to east, and assess their impact on the MOC by comparing the estimates with and without one specific change implemented. The time period over which the assessment is performed depends on the data availability, which ranges from 8 to 46 months (the maximum length of the observational record). All test runs have been conducted using the 30-day averaged data.

#### Updated velocity climatology for the Labrador shelf current (LSC)

The LSC is an unmeasured component at OSNAP West that flows southward at water depth shallower than ~300 m (see Fig. 1 for location). It carries the freshest and coldest waters in the subpolar region ($\theta \,\approx\,$ 0.8 °C and *S* ≈ 33.6), constituting a major component of the freshwater flux across the section^10^. In Lozier et al.^10^ monthly climatological velocities from a high-resolution (1/12°) regional ocean circulation model (FLAME) were used for representing the LSC at OSNAP. In the current calculations, we have used an ensemble-mean velocity climatology for the whole length of the record (46 months), which is derived from three ocean or ocean–sea-ice models and one ocean reanalysis (Supplementary Table 1). This is to reduce a potential transport bias from any specific model. In addition, World Ocean Atlas 2018 (WOA18) temperature^46^ and salinity^47^ climatology were used to replace WOA13.

The LSC transport shows very similar magnitudes among the four products with a shared seasonality that is strong in winter and weak in summer (not shown). The ensemble-averaged annual-mean LSC transport (−2.6 Sv) is slightly stronger than the transport in FLAME (−2.4 Sv). The LSC contains the lightest waters across the section and a stronger LSC transport (southward) causes a weaker transport in the upper MOC limb (northward). The comparison of the MOC estimates during 2014–2018 shows that using the ensemble-mean velocity climatology yields a reduction in the mean MOC by 0.2 Sv, compared to that using FLAME only.

#### Inclusion of the data from three RREX moorings

Three tall moorings that were part of the RREX array^48^ were deployed in the summer of 2015 right on the OSNAP line in the eastern Irminger basin (Fig. 1). They had been continuously measuring temperature, salinity and velocity throughout the water column for 2 years (from 29-Jun-2015 to 26-Jul-2017). The RREX moorings are situated between four existing OSNAP moorings, designed to capture the property and velocity structures of the northward-flowing Irminger Current (IC) above the western flank of the Reykjanes Ridge. The RREX mooring data were retrieved in the summer of 2017 and thus none of them were included in our first 21-month OSNAP time series. In the current calculations, we have incorporated the RREX mooring data in the property and velocity interpolations in the region whenever they are available.

To assess the impact on the flux estimate, we compared the transports calculated with and without the RREX mooring data during the 2-year RREX period. The comparisons show that using the RREX data yields a small increase in the mean IC transport (0.8 Sv or ~10% of the mean transport). Because the IC comprises waters in both the upper and lower MOC limbs, the corresponding impact on the MOC estimate is smaller. Over the 2015–2017 time period, the inclusion of the RREX mooring data results in an increase in the time-mean MOC by 0.2 Sv.

#### Modified reconstruction of the deep current in the Iceland basin

The deep current in the western Iceland basin contains the densest overflow waters in the subpolar North Atlantic flowing southward across the OSNAP East section. The deep current has a bottom velocity core above the eastern flank of the Reykjanes Ridge with a clear intrusion farther into the basin interior (Fig. 1b). Our modification of the calculation method is concerning the reconstruction of the interior transport, i.e., water deeper than ~2200 m and between 28.0 and 24.4°W (between the M2 and IB3 moorings; Supplementary Fig. 5). In Lozier et al.^10^ the bottom velocity field was derived based on the measurements from M2 and IB3 along with D4 in the following way: velocities from M2 and D4 were interpolated to fill the area between them, while geostrophic velocities were calculated between D4 and IB3 (Supplementary Fig. 5). In the current calculations, we have modified the method by disregarding the velocity interpolations and instead calculating the geostrophic flow between M2 and IB3 for the whole 2014–2018 time period. Because the M2 mooring is at a water depth (~2400 m) shallower than IB3 (~2800 m), we added the temperature and salinity measurements from the deepest instrument from D4 (at ~2600 m) to M2. It effectively minimizes the bottom triangle below the maximum common depth between the moorings M2 and IB3.

To assess the impact from this change, we compared the transport of bottom waters (*σ*_θ_ > 27.80 kg m^−3^) between M2 and IB3 for the whole 46-month time period. It shows that the new method yields a small reduction in the time-mean transport in the bottom layer (0.5 Sv) which leads to a reduction in the time-mean MOC by 0.2 Sv.

#### Updated objective analysis (OA) product

Away from the boundary arrays, temperature and salinity fields are created based primarily on the Argo and mooring data in the vicinity, using an OA technique. In Lozier et al.^10^ OA was performed on depth surfaces (OA_*depth*_), which inevitably tends to mix waters with different densities. In the current calculations, we have used OA gridded products where OA is performed on density surfaces (OA_*dens*_) to best preserve water properties. This updated product was used throughout the 2014–2018 time period. Input for the OA_*dens*_ comprises the Argo data, OSNAP mooring data as well as the WOA18 climatology. Note that OA_*dens*_ is limited to the upper water column owing to the limitation of the maximum sampling depth of Argo floats (2000 m). For the deeper layers, we use hydrographic data from the 2014 and 2016 summer OSNAP cruises. Density fields are calculated from gridded temperature and salinity fields using Gibbs Seawater Oceanographic Toolbox (version 3.06.11; http://www.teos-10.org/software.htm#1) of the International Thermodynamic Equation of Seawater-2010 (TEOS-10).

The OA_*dens*_ impact on the MOC estimate mainly arises from the use of density profiles for calculating the geostrophic flow in the glider survey domain above the Hatton–Rockall Plateau (Fig. 1), which is associated with the northward North Atlantic Current (NAC) transport in the upper MOC limb. To assess the impact, we calculated the transports with OA_*depth*_ and OA_*dens*_, respectively, for the whole 46-month observational period. The comparisons between those two estimates reveal an increase of ~1 Sv in the time-mean NAC transport in the region when using OA_*dens*_, with an increase in the time-mean MOC of 1.2 Sv.

#### Time-mean transport in the eastern Rockall Trough wedge

In the easternmost part of the OSNAP section, there is a strong northward-flowing shelf-break current (~1 Sv) in the eastern wedge of the Rockall Trough (Fig. 1). An upward-looking ADCP (acoustic Doppler current profiler) was mounted at the ~500 m water depth for capturing this wedge transport (Supplementary Fig. 6). However, due to multiple instrument losses, there has been only ~8 months of good data returned (30-Oct-2014 to 19-June-2015) during the whole 4-year deployment.

To accommodate this data loss, in Lozier et al.^10^ velocities from the nearest current meter mooring (RTEB1) to the ADCP were used to fill the wedge when the ADCP data are not available. To assess its impact (e.g., to what extent it captures the shelf-break current), the transports were calculated using the RTEB1 data following Lozier et al.^10^ during the 8-month time period when the ADCP data are available. Comparing it to the actual ADCP-derived transport suggests that using the RTEB1 data does not resolve the 1 Sv mean wedge transport. Because the transport is within the upper MOC limb, such an underestimation leads to an underestimation in the MOC of 0.8 Sv in the mean MOC during the same 8-month period. Thus, using the time-mean ADCP velocity for all the times when no ADCP data are returned leads to an increase in the time-mean MOC of 0.8 Sv.

#### Updated time-mean altimetry reference velocity

As part of the velocity calculations, the time-mean surface velocity over the observational record from satellite altimetry is used to provide the surface reference for calculating the absolute geostrophic velocities in the basin interior^10^. With the extension of the length of our records, we have now used 4-year mean altimetry velocity (23-Aug-2014 to 26-May-2018) in the current calculations. To assess the impact from this update, we compare the MOC estimates for the 2014–2018 time period using the time-mean surface velocity averaged over the first 21 months and over the whole 46 months, respectively. It shows a reduction in the time-mean MOC by 0.4 Sv with the 46-month mean altimetry reference. Note that in contrast to the other updates, the impact from the change in the reference velocity will be reduced with a longer record (i.e., when the change in the length of the record becomes relatively small compared to the length of record itself).

#### Impact on MOC estimates

The change in the mean MOC estimate is not a simple sum of all the changes caused by the individual updates listed above, because updates impact the velocity and MOC during certain times of the observational period.

Overall, the updates have changed the time-mean MOC during the first 21-month period from $14.9\,\pm\, 1.0\,{{{\rm{Sv}}}}$^10^ to $16.5\,\pm\, 1.0\,{{{\rm{Sv}}}}$, with negligible impact on the variability (*r* = 0.97). However, the difference in the mean MOC estimates is not statistically significant according to their standard errors.

### Water mass transformation rate

The time rate of the water mass transformation shown in Supplementary Fig. 3 is deduced from air-sea buoyancy fluxes by integrating surface density flux over the region where an isopycnal *σ* outcrops (e.g.,^49^). For each month, the transformation rate (*F*) at a given isopycnal can be derived as,

2 $$F({\sigma }^{\ast })\,=\,\frac{1}{\varDelta \sigma }\iint \left[-\frac{\alpha }{{C}_{P}}Q\,+\,\frac{S}{1\,-\,S}(E\,-\,P)\right]\prod (\sigma ){{{\rm{dxdy}}}}\,({{{\rm{Sv}}}}),$$

where

$$\varPi (\sigma )\,=\,\left\{\begin{array}{ll}1 & {{{\rm{for}}}}[\sigma \,-\,{\sigma }^{\ast }]\,\le\, \frac{\varDelta \sigma }{2}\\ 0 & {{{\rm{elsewhere}}}}.\end{array}\right.$$

In Eq. (2), *Q* is the net surface heat flux, *E* the evaporation rate, *P* the precipitation rate, *C*_*P*_ the specific heat capacity of seawater, *S* the surface salinity, *α* and *β* the thermal expansion and haline contraction coefficients, respectively. To calculate, we use $\varDelta \sigma \,=\,0.2\,{{{\rm{kg}}}}\,{m}^{-3}$. *Q*, *E*, and *P* are obtained by averaging the monthly NCEP/NCAR^50^ and ERA5 surface flux products to the 1/4° ERA5 grids. The outcropping area of each σ for each month is determined from the EN4 gridded subsurface salinity^51^ and ERA5 sea surface temperature.

Uncertainty for the monthly *F* is obtained as the ensemble standard error by taking into account the estimates using a variety of Δσ with surface buoyancy fluxes from either NCEP/NCAR^50^ or ERA5. Uncertainty in the time-mean *F* is obtained by combining the uncertainty in individual months randomly following standard error propagation theory.

### Argo data

Temperature and salinity sampled by profiling floats as part of the Argo program (http://www.argo.ucsd.edu) are used to produce density profiles and to calculate the UNADW layer thickness in the Labrador and Irminger basin interiors. Argo data between 2014 and 2018 were downloaded by the U.S. Global Ocean Data Assimilation Experiment (USGODAE) Argo Data Assembly Center. We used delayed-mode data with quality flag of 1 (good) or 2 (probably good) and rejected all problematic Argo profiles according to the Argo floats gray list (www.nodc.noaa.gov/argo/grey_floats.htm). Typically, there were 40 and 35 profiles in the Labrador and Irminger basin interior, respectively, each month during 2014–2018. Here the Labrador basin interior is defined as the region north of the OSNAP West line and deeper than 3000 m; the Irminger basin interior is the region north of the OSNAP East line and deeper than 2000 m. All profiles were linearly interpolated onto a uniform vertical grid with 20 m intervals, and then used for calculating layer thickness. The interior thickness is computed as the average of the 20% largest values of all available profiles.

### Statistical analysis

Lagged cross-correlations between two daily time series A and B are performed on data with daily means and the linear trend removed. The daily means are constructed by averaging the values of the same day of the year for all years.

The uncertainty in the correlations is examined using the 95% significance level obtained as $1.96/\sqrt{{N}_{{{{\rm{eff}}}}}}$. *N*_eff_ is the effective number of degrees of freedom that takes into account autocorrelation (e.g.,^52^):

3 $${N}_{{{{\rm{eff}}}}}\,=\,N\frac{1\,-\,{r}_{A}{r}_{B}}{1\,+\,{r}_{A}{r}_{B}},$$

where *N* is the number of observations, *r*_A_ and *r*_B_ are the lag 1 autoregressive autocorrelation coefficient of the two variables, respectively.

As an alternative approach, we follow McCarthy et al.^53^ and evaluate the uncertainty in the correlations by calculating *p* values from the *t*-statistic,

4 $$T\,=\,r\sqrt{\frac{{N}_{{{{\rm{eff}}}}}\,-\,2}{1\,-\,{r}^{2}}},$$

where *r* is the obtained cross-correlation coefficient.

The statistical significance of the difference between two independent estimates is obtained following a common practice, i.e., by comparing the standard errors of the two sets (e.g.,^54^). If the gap between the two standard error bars equals the average of them, it indicates $p\,\approx\, 0.05$.

The significance of the seasonal variations in the MOC is tested using one-way ANOVA (analysis of variance), by calculating *p* values from the *f*-statistic,

5 $$F\,=\,\frac{{{{\rm{SSB}}}}/(k\,-\,1)}{{{{\rm{SSE}}}}/(N\,-\,k)}.$$

In Eq. (5), SSB is the variance between months,

6 $${{{\rm{SSB}}}}\,=\,\mathop{\sum }\limits_{j\,=\,1}^{k}{n}_{j}{({\bar{x}}_{j}\,-\,\bar{x})}^{2},$$

where *k* is the number of months in the climatology, ${\bar{x}}_{j}$ is the mean for each month, *n*_*j*_ is the number of values used to calculate each mean, and $\bar{x}$ is the overall mean. SSE is the variance within all months,

7 $${{{\rm{SSE}}}}\,=\,\mathop{\sum }\limits_{i\,=\,1}^{N}{({x}_{i}\,-\,{\bar{x}}_{j})}^{2},$$

where *x*_*i*_ is individual monthly values, and *N* is the total number of months.

The integral time scale of the MOC at OSNAP is calculated from the autocorrelation function of the daily time series^55^, which is 6 days and 13 days for OSNAP West and East, respectively. Therefore, there is one independent observation every month.

Using Eq. (5), we obtained *F* = 1.31, *p* = 0.52 for OSNAP West, and *F* = 1.73, *p* = 0.24 for OSNAP East.

We use the square of correlation coefficient (*r*^2^) for evaluating how much the total MOC variance can be explained by a reconstructed MOC. That is, *r*^2^ value means that a linear regression of the reconstructed MOC on the actual MOC explains $({r}^{2} \,\cdot\, 100 \% )$ of the total variance in the latter (e.g.,^55^).

## Supplementary information

Supplementary Information

## Data Availability

The 2014–2018 OSNAP MOC time series is available in SMARTech Repository^56^. Calibrated and quality-controlled data from moored instruments and gliders were generated by each participating group and are available in designated repositories (www.o-snap.org).

## Code availability

The code used to compute the OSNAP time series, and water mass layer thickness anomaly can be accessed upon request to F.L.
