# Author Correction: Subpolar North Atlantic western boundary density anomalies and the Meridional Overturning Circulation

**DOI:** 10.1038/s41467-022-28397-3

**Published:** 2022-02-02

**Authors:** F. Li, M. S. Lozier, S. Bacon, A. S. Bower, S. A. Cunningham, M. F. de Jong, B. deYoung, N. Fraser, N. Fried, G. Han, N. P. Holliday, J. Holte, L. Houpert, M. E. Inall, W. E. Johns, S. Jones, C. Johnson, J. Karstensen, I. A. Le Bras, P. Lherminier, X. Lin, H. Mercier, M. Oltmanns, A. Pacini, T. Petit, R. S. Pickart, D. Rayner, F. Straneo, V. Thierry, M. Visbeck, I. Yashayaev, C. Zhou

**Affiliations:** 1 State Key Laboratory of Marine Environmental Science & College of Ocean and Earth Sciences, Xiamen University, Xiamen, China; 2 School of Earth and Atmospheric Sciences, Georgia Institute of Technology, Atlanta, GA USA; 3 National Oceanography Centre, Southampton, UK; 4 Woods Hole Oceanographic Institution, Woods Hole, MA USA; 5 Scottish Association for Marine Science, Oban, UK; 6 NIOZ Royal Netherlands Institute for Sea Research, Texel, Netherlands; 7 Department of Physics and Physical Oceanography, Memorial University, St. John’s, NL Canada; 8 Fisheries and Oceans Canada, Institute of Ocean Sciences, Sidney, BC Canada; 9 Fisheries and Oceans Canada, Northwest Atlantic Fisheries Centre, St. John’s, NL Canada; 10 Scripps Institution of Oceanography, UCSD, La Jolla, CA USA; 11 School of Geosciences, Edinburgh University, Edinburgh, UK; 12 Department of Ocean Sciences, University of Miami, Miami, FL USA; 13 GEOMAR Helmholtz Centre for Ocean Research Kiel, Kiel, Germany; 14 Univ. Brest, Ifremer, CNRS, IRD, Laboratoire d’Océanographie Physique et Spatiale, Plouzané, France; 15 Frontier Science Center for Deep Ocean Multispheres and Earth System and Physical Oceanography Laboratory, Ocean University of China, Qingdao National Laboratory for Marine Science and Technology, Qingdao, China; 16 CNRS, Laboratoire d’Océanographie Physique et Spatiale, Plouzané, France; 17 Bedford Institute of Oceanography, Dartmouth, NS Canada

**Keywords:** Ocean sciences, Physical oceanography

Correction to: *Nature Communications* 10.1038/s41467-021-23350-2, published online 24 May 2021.

The original version of this Article contained an error in the caption of Figure 2, in which the total Ekman transport was incorrectly listed as −0.7 ± 0.01 Sv. The correct value is −1.5 ± 0.02 Sv. The corrected article also has an updated data product link in Reference 56. These have been corrected in both the PDF and HTML versions of the Article.

