# Supplementary material for: Subpolar North Atlantic western boundary density anomalies and the Meridional Overturning Circulation
Source: Nat Commun. 2021 May 24;12:3002. doi: 10.1038/s41467-021-23350-2 (PMC8144605; doi:10.1038/s41467-021-23350-2)
Supplement: Supplementary file 1 — Supplementary Information [file 41467_2021_23350_MOESM1_ESM.pdf]

SUPPLEMENTARY INFORMATION

Subpolar North Atlantic western boundary density anomalies and the Meridional Overturning Circulation

F. Li[1,2*], M.S. Lozier[2*], S. Bacon[3], A.S. Bower[4], S.A. Cunningham[5], M.F. de Jong[6], B. deYoung[7], N. Fraser[5], N. Fried[6], G. Han[8], N.P. Holliday[3], J. Holte[9], L. Houpert[3], M.E. Inall[5,10], W.E. Johns[11], S. Jones[5], C. Johnson[5], J. Karstensen[12], I.A. LeBras[4,9], P. Lherminier[13], X. Lin[14], H. Mercier[15], M. Oltmanns[12], A. Pacini[4], T. Petit[2], R.S. Pickart[4], D. Rayner[3], F. Straneo[9], V. Thierry[13], M. Visbeck[12], I. Yashayaev[16], C. Zhou[14]

[1] State Key Laboratory of Marine Environmental Science & College of Ocean and Earth Sciences, Xiamen University, Xiamen, China.
[2] School of Earth and Atmospheric Sciences, Georgia Institute of Technology, Atlanta, Georgia, USA.
[3] National Oceanography Centre, Southampton, UK.
[4] Woods Hole Oceanographic Institution, Woods Hole, Massachusetts, USA.
[5] Scottish Association for Marine Science, Oban, UK.
[6] NIOZ Royal Netherlands Institute for Sea Research, Texel, Netherlands.
[7] Department of Physics and Physical Oceanography, Memorial University, St. John’s, Newfoundland, Canada.
[8] Fisheries and Oceans Canada, Northwest Atlantic Fisheries Centre, St. John’s, Newfoundland, Canada. Fisheries and Oceans Canada, Institute of Ocean Sciences, Sidney, British Columbia, Canada.
[9] Scripps Institution of Oceanography, UCSD, La Jolla, California, USA.
[10] School of Geosciences, Edinburgh University, Edinburgh, UK.
[11] Department of Ocean Sciences, University of Miami, Miami, Florida, USA.
[12] GEOMAR Helmholtz Centre for Ocean Research Kiel, Kiel, Germany.
[13] Univ. Brest, Ifremer, CNRS, IRD, Laboratoire d'Océanographie Physique et Spatiale, Plouzané, France.
[14] Frontier Science Center for Deep Ocean Multispheres and Earth System and Physical Oceanography Laboratory, Ocean University of China and Qingdao National Laboratory for Marine Science and Technology, Qingdao, China.
[15] CNRS, Laboratoire d'Océanographie Physique et Spatiale, Plouzané, France.
[16] Bedford Institute of Oceanography, Dartmouth, Nova Scotia, Canada.

*Correspondence to: F.L. (feili.li@xmu.edu.cn); M.S.L. (susan.lozier@gatech.edu)

Supplementary Figures

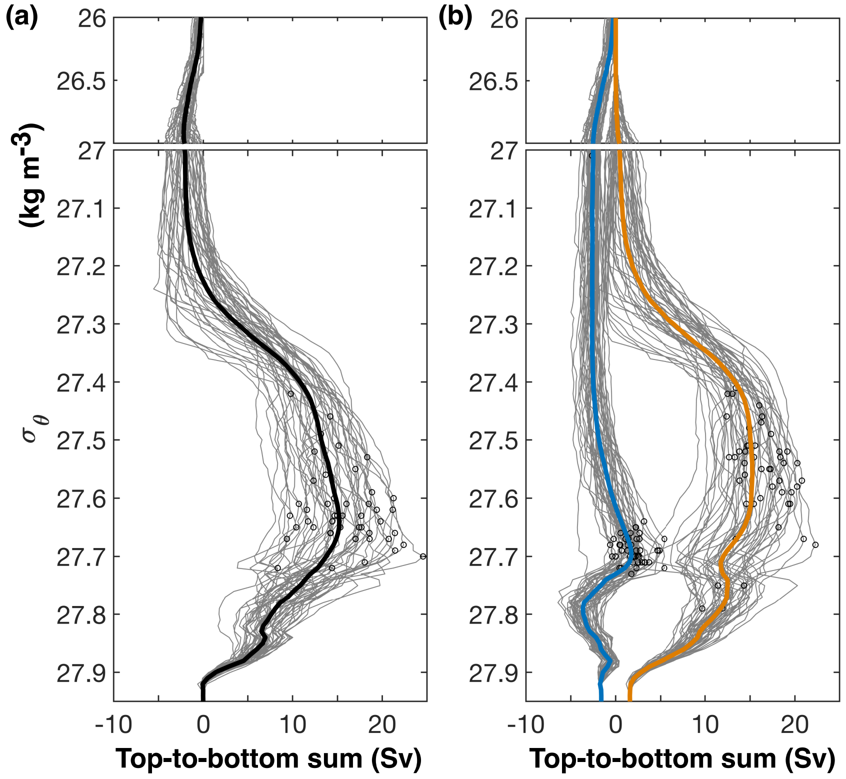

Supplementary Fig. 1. Overturning streamfunction. Streamfunction in density space across **(a)** the full array and, and **(b)** at OSNAP West (blue) and East (red) separately. Gray lines are streamfunction during individual 30-day time intervals. The potential densities corresponding to the maximum of the streamfunction (σ_{MOC}) are indicated by circles. In the mean, those potential densities across the subsections (27.70 kg m^{-3} for OSNAP West and 27.55 kg m^{-3} for OSNAP East) are slightly different from that across the full array (27.65 kg m^{-3}). As a result, the MOC across the full array is smaller than the sum of the OSNAP West MOC and OSNAP East MOC, due to the cancellation of the currents around Greenland [1].

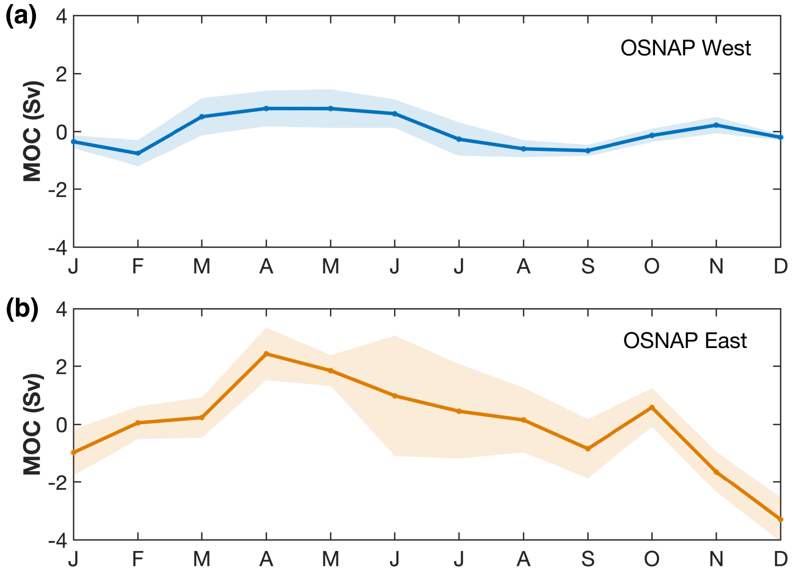

Supplementary Fig. 2. Seasonal overturning changes. Monthly composite of the MOC anomalies at **(a)** OSNAP West and **(b)** East. Shading represents ±1 standard error of the monthly estimates, which is obtained by dividing the standard deviation by $\sqrt{n}$, where n is the number of years (assuming each month from separate years is independent).

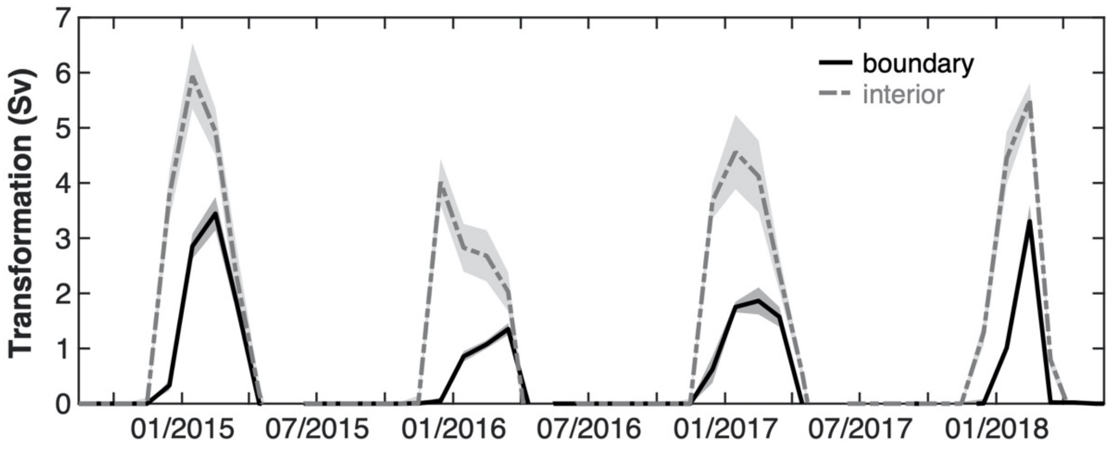

Supplementary Fig. 3. Labrador Sea water mass transformation. Air-sea buoyancy flux derived water mass transformation rate across the isopycnal of 27.70 kg m^{-3} in the boundary and interior of the Labrador Sea. Shading indicates the uncertainty in each monthly estimate (Methods). The interior (boundary) is defined as the area with water deeper (shallower) than 3000m and northwest of the OSNAP West line.

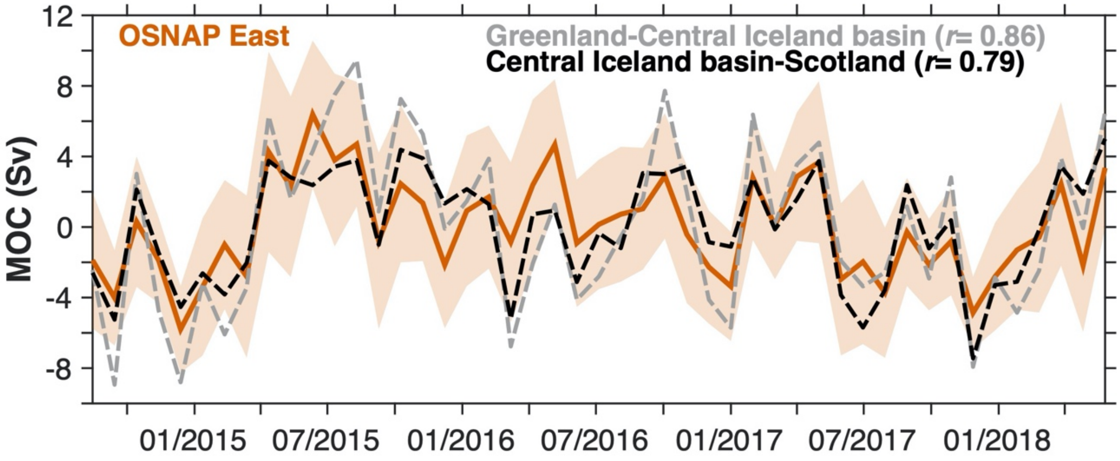

Supplementary Fig. 4. Overturning anomalies across OSNAP East. MOC derived from the full OSNAP East array (red line; shading indicates uncertainty in each 30-day estimate obtained from Monte Carlo simulations [1]) compared to the reconstructed MOC. The reconstruction is obtained from the time-varying velocities and densities at the western (eastern) part of the array, along with the time-mean data at the remaining eastern (western) part of the array. 'Central Iceland basin' is the location at the OSNAP line that approximately separates the upper and lower limbs of MOC (see Fig. 1b for the location ~2600 m). Numbers in parentheses are the maximum correlation coefficients between the MOC from the full OSNAP East array and the reconstructed time series, which are at the zero lag and are significant at the 95% level (Methods).

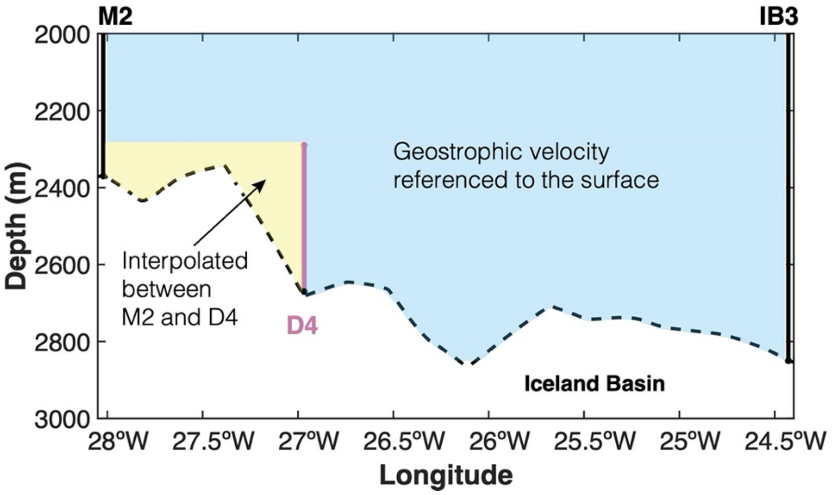

Supplementary Fig. 5. OSNAP moorings M2, IB3, D4 in the Iceland basin. Vertical lines indicate the depth range of the mooring measurements. The M2 and IB3 are tall moorings that extended all the way up to the sea surface (not displayed). The dashed line indicates topography.

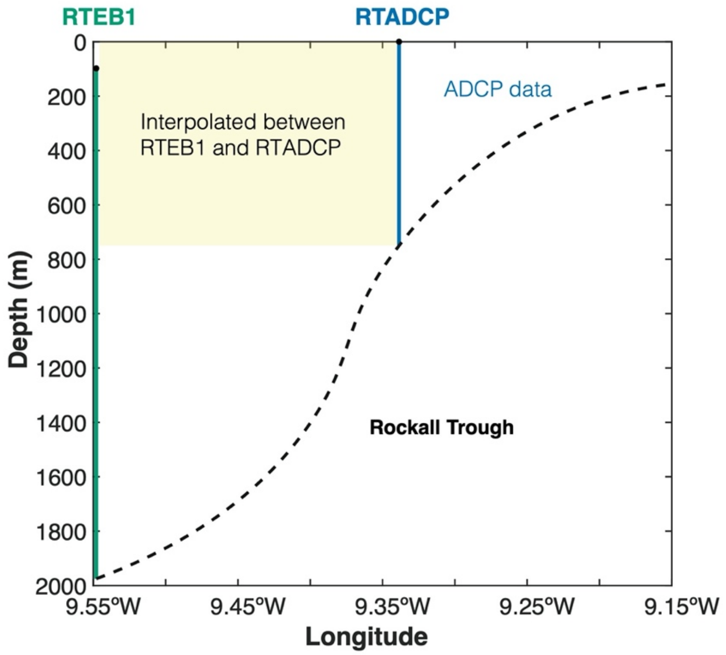

Supplementary Fig. 6. OSNAP moorings RTEB1 and RTADCP in the eastern Rockall Trough. Vertical lines indicate the range of direct velocity measurements from the moorings. ADCP data are filled into the eastern wedge when they are available. The dashed line indicates topography.

Supplementary Table 1. Velocity climatology for the Labrador shelf current. Model output used for creating the ensemble-mean velocity climatology above the Labrador shelf. The transports are mean plus/minus standard deviation.

**Name**	**Ocean model**	**Sea-ice model**	**Nominal horizontal resolution**	**Vertical levels (*coordinate*)**	**Reference**	**Time period for averaging**	**Annual-mean LSC transport**
OPA	NEMO-OPA	LIM2	7 km (~1/12°)	46 (*z*)	[2]	2014-2017	–2.6 ± 0.3 Sv
ANHA12	NEMO	LIM2	1/12°	50 (*z*)	[3,4]	2014-2018	–2.9 ± 0.6 Sv
FLAME	MOM2.1	n/a	1/12°	45 (*z*)	[5,6]	1990-2004	–2.4 ± 0.6 Sv
GLORYS12v1	NEMO3.1	LIM2	1/12°	50 (*z*)	[7]	2014-2018	–2.3 ± 0.3 Sv

Supplementary References

1. Lozier, M. S. *et al.* A sea change in our view of overturning in the subpolar North Atlantic. *Science* **363**, 516-521, doi:10.1126/science.aau6592 (2019).
2. Han, G. Q., Ma, Z. M. & Chen, N. Ocean climate variability off Newfoundland and Labrador over 1979-2010: A modelling approach. *Ocean Modelling* **144**, doi:10.1016/j.ocemod.2019.101505 (2019).
3. Grivault, N., Hu, X. M. & Myers, P. G. Impact of the Surface Stress on the Volume and Freshwater Transport Through the Canadian Arctic Archipelago From a High-Resolution Numerical Simulation. *Journal of Geophysical Research-Oceans* **123**, 9038-9060, doi:10.1029/2018jc013984 (2018).
4. Hu, X. M., Sun, J. F., Chan, T. O. & Myers, P. G. Thermodynamic and dynamic ice thickness contributions in the Canadian Arctic Archipelago in NEMO-LIM2 numerical simulations. *Cryosphere* **12**, 1233-1247, doi:10.5194/tc-12-1233-2018 (2018).
5. Eden, C. & Willebrand, J. Mechanism of interannual to decadal variability of the North Atlantic circulation. *Journal of Climate* **14**, 2266-2280 (2001).
6. Böning, C. W., Scheinert, M., Dengg, J., Biastoch, A. & Funk, A. Decadal variability of subpolar gyre transport and its reverberation in the North Atlantic overturning. *Geophysical Research Letters* **33**, L21S01, doi:10.1029/2006GL026906 (2006).
7. Lellouche, J. M. *et al.* Recent updates to the Copernicus Marine Service global ocean monitoring and forecasting real-time 1/12° high-resolution system. *Ocean Sci.* **14**, 1093-1126, doi:10.5194/os-14-1093-2018 (2018).
